# Multivariate strategy for the sample selection and integration of multi-batch data in metabolomics

**DOI:** 10.1007/s11306-017-1248-1

**Published:** 2017-08-24

**Authors:** Izabella Surowiec, Erik Johansson, Frida Torell, Helena Idborg, Iva Gunnarsson, Elisabet Svenungsson, Per-Johan Jakobsson, Johan Trygg

**Affiliations:** 1 Computational Life Science Cluster (CLiC), Department of Chemistry, Umeå University, 901 81 Umeå, Sweden; 2 Sartorius Stedim Data Analytics AB, 907 19 Umeå, Sweden; 3 Rheumatology Unit, Department of Medicine, Solna, Karolinska Institutet, Karolinska University Hospital, 171 76 Stockholm, Sweden

**Keywords:** OPLS, Metabolomics, Multi-batch analysis, Representative sample selection

## Abstract

**Introduction:**

Availability of large cohorts of samples with related metadata provides scientists with extensive material for studies. At the same time, recent development of modern high-throughput ‘omics’ technologies, including metabolomics, has resulted in the potential for analysis of large sample sizes. Representative subset selection becomes critical for selection of samples from bigger cohorts and their division into analytical batches. This especially holds true when relative quantification of compound levels is used.

**Objectives:**

We present a multivariate strategy for representative sample selection and integration of results from multi-batch experiments in metabolomics.

**Methods:**

Multivariate characterization was applied for design of experiment based sample selection and subsequent subdivision into four analytical batches which were analyzed on different days by metabolomics profiling using gas-chromatography time-of-flight mass spectrometry (GC–TOF–MS). For each batch OPLS-DA^®^ was used and its p(corr) vectors were averaged to obtain combined metabolic profile. Jackknifed standard errors were used to calculate confidence intervals for each metabolite in the average p(corr) profile.

**Results:**

A combined, representative metabolic profile describing differences between systemic lupus erythematosus (SLE) patients and controls was obtained and used for elucidation of metabolic pathways that could be disturbed in SLE.

**Conclusion:**

Design of experiment based representative sample selection ensured diversity and minimized bias that could be introduced at this step. Combined metabolic profile enabled unified analysis and interpretation.

**Electronic supplementary material:**

The online version of this article (doi:10.1007/s11306-017-1248-1) contains supplementary material, which is available to authorized users.

## Introduction

Increased availability of modern high-throughput ‘omics’ technologies resulted in the potential for generating massive amounts of chemical data. At the same time, availability of large cohorts of samples with related metadata has increased in recent years, providing scientists with extensive and well-described material for studies. These developments place additional requirements on study planning and execution, for which selection of relevant samples, extraction and integration of useful information from obtained data are important (Bictash et al. 2010; McCarthy et al. 2008).

When planning to analyze samples from large cohorts, the cost of analysis and/or the potential for sparing samples for future research are critical issues to consider. These constraints usually result in the need for representative sample selection. Representative samples are the samples whose characteristics or inferences from their analysis approximate population values and as such provide similar conclusions as would be obtained from the investigation of all available samples. While random sampling strategies are commonly used, lowering of possible selection bias can be obtained by application of the supervised sample selection approaches. Matched pairs comprise a reliable method for use when there are few clinical parameters (e.g. gender, age, BMI) to consider (Hulley 2013; Wuolikainen et al. 2016), but are inadequate approach when tens to hundreds of clinical and personal parameters describing the samples (sample descriptors) are available. As samples and their descriptors create a multivariate data set, they can form the basis for sample selection using a multivariate characterization approach (Eriksson et al. 2013). Multivariate characterization is an essential application of principal component analysis (PCA), which is a basis for representative sample selection with design-based approaches. Multivariate characterization creates a low-dimensional map from the study samples and their descriptors using PCA. PCA scores adequately summarize the properties of the study samples. The notable feature of the scores is that they are mathematically independent of each other (orthogonal) and usually limited in number (between two and four). Multivariate characterization is especially useful for quantifying changes in discrete multi-level factors (factors that can take only finite, higher than two, number of values), and has been successfully used in several fields for selecting sets of compounds and substituents representative for the question of the study, e.g. in synthetic organic chemistry (Carlson and Nordahl 1993), medicinal chemistry (Eriksson et al. 2004; Giraud et al. 2000), environmental chemistry (Ramos et al. 1997; Tysklind et al. 1995) and microbiology (Marvanova et al. 2001).

When data complexity is reduced with multivariate characterization, several approaches for representative sampling from a multivariate space can be used. One possible method, the space-filling design, targets even distribution of the design points throughout the space of interest (Thysell et al. 2012). Other possible methods involve statistical, experimental design schemes such as factorial or fractional factorial designs (Box et al. 1978), D-optimal designs (deAguiar et al. 1995), or the onion design (Olsson et al. 2004). Any set of samples selected according to an appropriate multivariate design will have the best diversity and spread among the latent variables that can be achieved with the available samples. Multivariate characterization can also be used to divide selected samples into analytical batches if all samples cannot be analyzed concurrently (e.g. at the same day), which is inevitable with larger cohorts. Sample division into representative batches ensures a controllable analysis and enables treatment of each individual batch as an independent study (Thysell et al. 2012).

Integration of data from these representative analytical batches corresponds in the classical statistics to the general problem of randomized block designs and application of blocking factors to reduce variation not related to the studied effect (Box et al. 1978). It presents a big challenge especially for untargeted profiling experiments (like metabolomics, proteomics, and transcriptomics) (Leek et al. 2010), because, contrary to targeted approaches, compounds subjected to profiling methods are not absolutely quantified, and data interpretation is based on relative comparison of compound levels. These levels are influenced by instrument drift and other analytical errors that are inevitably part of each analysis, and which can introduce bias and hinder provision of biologically relevant information (Burton et al. 2008). Within particular analysis analytical drift can be removed by data normalization, with application of different scaling factors (Cairns et al. 2008; Wang et al. 2003), internal standards (Redestig et al. 2009; Sysi-Aho et al. 2007), optimally selected endogenous compounds (De Livera et al. 2012; Warrack et al. 2009) or quality control samples (De Livera et al. 2015; Fernandez-Albert et al. 2014). Several methods were also presented for correction of peak intensity drift in multi-batch metabolomics studies, with the batch-corrected data being subsequently concatenated and analyzed (Draisma et al. 2010; Wang et al. 2013). The main advantage of such approach is easier data handling and increased power of statistical analysis of the obtained data matrix compared to analysis of separate datasets. Batch correction methods have big potential in metabolomics studies, which still has to be verified for experiments performed in large time intervals and for integration of data obtained from different research groups. New solutions for combined data analysis are needed for the situations where drift removal approaches are not applicable or not sufficiently effective. One possibility would be to, instead of combining data sets, concatenate study-relevant results obtained from analysis of separate batches/studies.

Statistical evaluation of metabolomics data can be achieved by application of univariate or multivariate methods such as support vector machines (SVM) (Mahadevan et al. 2008), neural networks (Taylor et al. 2002), principal components analysis (PCA) (Jackson 2003), cluster analysis (Li et al. 2009), partial least squares (PLS) (Wold et al. 2001) or orthogonal PLS (OPLS) (Trygg and Wold 2002). OPLS separates the systematic variation in the metabolite data into two parts, one part that is correlated (predictive) to the response (Y, e.g. class belonging) and one part that is uncorrelated (orthogonal). The main benefits include model transparency and interpretation. In OPLS, relevant information about the metabolic profile is stored in a correlation-scaled predictive loading vector (p(corr)), with p(corr) values ranging from −1.0 to 1.0. A high absolute p(corr) value indicates that a given metabolite is more abundant in one group [e.g. disease, positive p(corr) value] than in another [e.g. controls, negative p(corr) value]. The p(corr) vector values are independent on the scaling of the data. These properties allow p(corr) vectors to be directly comparable between studies, as long as the same variables were included in the OPLS models (Wiklund et al. 2008). OPLS p(corr) vectors were already applied for example in evaluation of treatment effects (Stenlund et al. 2009).

**Fig. 1:**
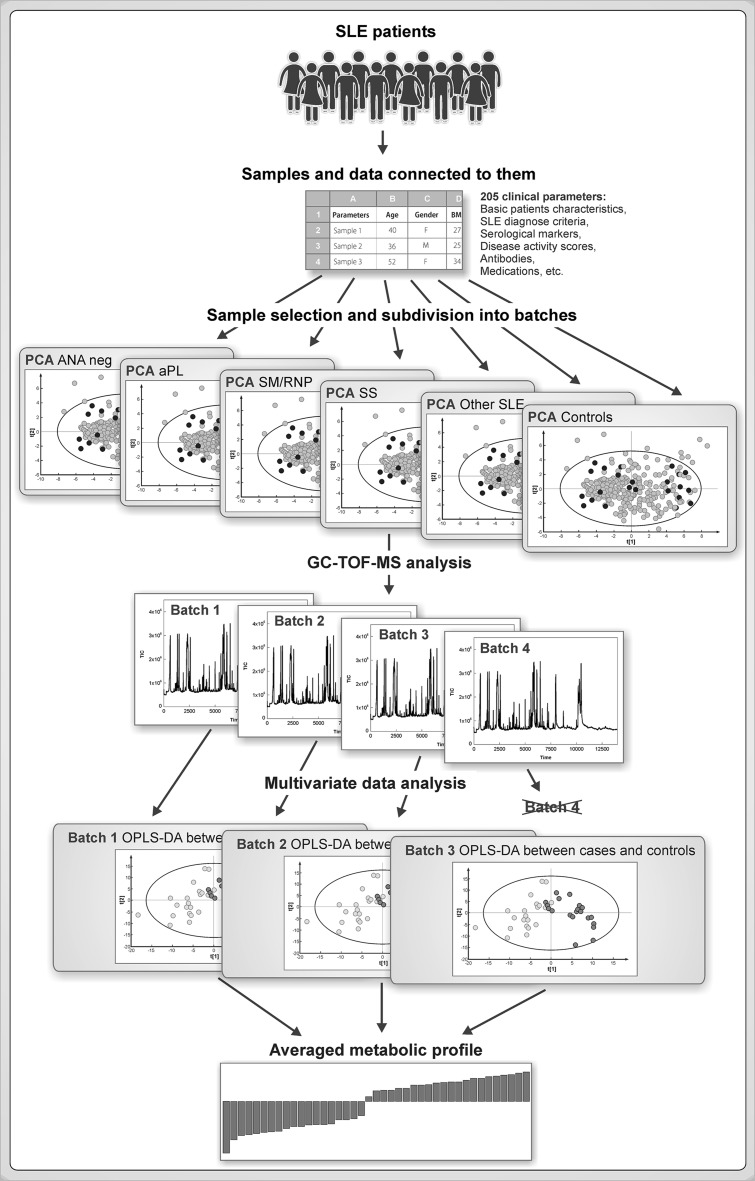
Overview of the experimental strategy applied in this study comprising of the following steps: (1) representative selection of samples from each of the studied sample classes (SLE subgroups) based on available clinical and personal sample descriptors with the application of multivariate characterization and DOE approach; (2) application of the same strategy for subdivision of samples in the representative analytical batches; (3) chemical analysis of samples; (4) OPLS modeling of samples in each batch respectively to the question of the study; (5) averaging of the OPLS p(corr) vectors from all batches to obtain combined metabolic profile

Herein we present a strategy based on multivariate methodology for sample selection and integration of experimental data from multi-batch experiments in metabolomics. The strategy is summarized at Fig. 1 and comprises of the following steps: (1) representative selection of samples from each of the studied sample classes based on available clinical and personal sample descriptors with the application of multivariate characterization and DOE approach; (2) application of the same strategy for subdivision of samples in the representative analytical batches; (3) chemical analysis of samples; (4) OPLS modeling of samples in each batch respectively to the question of the study; (5) averaging of the OPLS p(corr) vectors from all batches to obtain combined metabolic profile. The methodology is presented using a clinical study of SLE as an example.

## Experimental

### Patients

Systemic lupus erythematosus (SLE) is a chronic autoimmune disease predominantly diagnosed in women with very diverse manifestations as well as disease onset. It is a connective-tissue disorder characterized by immunological abnormalities and the involvement of a variety of organ systems, like skin, joints, kidneys, heart and the central nervous system (D’Cruz et al. 2007). It is currently defined by Systemic Lupus International Collaborating Clinic, or SLICC-criteria (Petri et al. 2012). The criteria involve clinical assessment supported by immunological manifestations criteria, including detection of various autoantibodies. Because SLE is a very heterogeneous condition presenting diverse manifestations from almost all organ systems it is often mistaken for other diseases and the biomarkers used today to diagnose and to monitor disease activity are far from perfect with respect to sensitivity and specificity (Liu and Ahearn 2009). The lack of good disease markers undermines also efforts to monitor and evaluate the effects of novel therapeutics in clinical trials. All this drives the search for new diagnostic tools and treatments, which could also bring increased understanding of the underlying disease factors.

Patients that fulfilled four or more classification criteria for SLE were included in the Karolinska Institutet SLE cohort. A total of 320 SLE patients and 320 population controls were enrolled at the time of study initiation. Controls were population-based individuals identified through the population registry and individually matched to each patient according to age, gender and region of living. The only exclusion criterion was a SLE diagnosis. Patients and controls were evaluated in person by a rheumatologist. Extensive personal and basic clinical data were collected together with serological and urinary markers, kidney parameters, medications, disease activity scores, genetic factors, antibody levels, treatments, environmental exposures and information about previous and concurrent diseases and SLE manifestations. Fasting ethylenediaminetetraacetic acid (EDTA) plasma samples were collected from all study participants according to standardized protocols and stored at −80 °C. All participants gave written informed consent to participate in the study which was approved by the ethical board at Karolinska University Hospital, Sweden.

### Sample selection and subdivision into batches

In this paper a multivariate approach was used to select a subset of samples from the entire Karolinska SLE cohort. Two hundred and five sample descriptors formed the basis for sample selection with the multivariate characterization approach. Since SLE predominantly affects women, only female samples were used in this study. The cohort was divided into five subgroups based on the patients’ antibody profiles. This division was inspired by previous observations (Artim-Esen et al. 2014; To and Petri 2005) that lupus patients can be divided into groups that differ by symptoms and prognosis. Patients were assigned to the following SLE subgroups: antinuclear antibody negative (ANA neg, patients in this group were negative for antibodies investigated at the time of sampling); antiphospholipid positive SLE [aPL, patients that tested positive for at least two of the following antibodies: anti-cardiolipin antibodies (aCL IgG, aCL IgM and aCL IgA) and β-2-glycoprotein-1 antibodies (or apolipoprotein H antibodies, B2GP1IgG, B2GP1IgM, and B2GP1IgA)]; anti-Sm/anti-RNP antibodies [SM/RNP, patients that tested positive for at least two of the following: anti-Smith antibodies (Sm) and/or anti-ribonucleoprotein antibodies (RNP A, RNP 68)]; Sjögren’s syndrome antigens (A/B) positive [SS, patients that tested positive for at least two antibodies against Sjögren’s syndrome A antigen (Ro60 or Ro52) and Sjögren’s syndrome B antigen (La)]; and other SLE (patients in this group did not fit into any of the previous groups or overlapped between two or more of them).

For each group we used PCA modelling on available personal and clinical parameters to summarize samples into a low-dimensional hyperplane, visualized as a two-dimensional score scatter plot. A full two-level factorial experimental design was applied to the PCA score plot, with five samples selected from each of the four corners of the design and three from the design’s center point (see Fig. S1). This procedure was repeated for each SLE subgroup and the control group, resulting in the selection of 23 samples from each subgroup. Only 22 samples were available from the aPL positives subgroup, and all were included for analysis. Sample selection produced 114 SLE samples and 23 controls for the study. This PCA-based sample selection procedure was repeated for sub-division of samples into four analytical batches analyzed at different days, with five to six samples from each SLE-subgroup and controls included in each batch.

### GC–TOF–MS analysis and data processing

Plasma samples were extracted, derivatized, and analyzed using GC–TOF–MS as previously described (Jiye et al. 2005) and as summarized in the Supplementary Material. Non-processed files from GC–TOF–MS analysis were then exported in NetCDF format to a MATLAB-based in-house script where all data pre-treatment procedures such as baseline correction, chromatogram alignment, and peak deconvolution were performed. Metabolite identification was implemented within the script and was based on the comparison of retention index (RI) values and MS spectra of the deconvoluted metabolites with the ones from the in-house mass spectra library established at the same instrument by the Swedish Metabolomics Centre (Umeå, Sweden) [Level 1 identification according to MSI (Salek et al. 2013)]. Seventy-three metabolites were identified using this procedure. Peak areas obtained were normalized using the areas from eleven internal standards that eluted during the entire chromatograph according to the following procedure. A PCA model with unit variance scaling [UVN scaling; for each variable (metabolite) the standard deviation (*s*_*k*_) is calculated and then each value for this variable is multiplied by 1/*s*_*k*_, average is not subtracted], and using peak areas of internal standards, was calculated. The t1-score vector from this model was used for normalization of the data which was done by dividing the all peak areas in each sample by its corresponding t1-score value (Redestig et al. 2009).

### Statistical analysis of the metabolomics data

All multivariate modelling was performed using SIMCA version 14 (MKS Data Analytics Solution, Umeå, Sweden). PCA was used for the sample selection procedure, and OPLS-DA^®^ was used to elucidate the metabolomics differences between various groups of subjects. Column centering and scaling to unit variance was used for all models, and model significance was found by means of sevenfold cross-validation. Number of model components was evaluated by a cross-validation procedure, but no more than two components were selected to avoid over-fitting.

For each batch run order effect was checked by calculating a 1 + 1 OPLS model with all metabolite signals as X variables and sample run order as Y variable. For batch 4 high run order effect was observed (20%) as compared to other batches (12, 8 and 8% for batch 1, 2 and 3 respectively), which could be explained by the visible, uncontrollable drop in instrument sensitivity during the analysis.

Metabolites significant for the OPLS-DA models between SLE and controls were identified using confidence intervals calculated by multiplying the jackknife standard errors by the t-value (α = 0.05, two-tailed) corresponding to the N − 1 degrees of freedom, where N is the number of the cross-validation groups. Jackknifing is a method for finding the precision of an estimate, by iteratively keeping out parts of the underlying data, making estimates from the subsets and comparing these estimates (Efron and Gong 1983). The p(corr) values from each batch’s OPLS-DA model for metabolites that had the same sign of p(corr) vector in all batches were averaged to obtain an average/combined metabolic profile characterizing SLE versus controls. The average standard error for each metabolite in the combined profile was calculated according to the following formula:

$${\text{S}}{{\text{E}}_{{\text{avg}}}}={\left( {{{\left( {{\text{S}}{{\text{E}}_1}^{2}+{\text{S}}{{\text{E}}_2}^{2}+{\text{S}}{{\text{E}}_3}^{2}} \right)} \mathord{\left/ {\vphantom {{\left( {{\text{S}}{{\text{E}}_1}^{2}+{\text{S}}{{\text{E}}_2}^{2}+{\text{S}}{{\text{E}}_3}^{2}} \right)} 3}} \right. \kern-0pt} 3}} \right)^{1/2}}$$

where SE_avg_ is the average standard error obtained from jackknife standard errors from each batch (SE_1_ − SE_3_). Results were presented as average p(corr) vector, with average confidence interval defining the significance of the metabolite in this vector. The average confidence interval was calculated by multiplying average standard error via the t-value (α = 0.05, two-tailed) corresponding to the N − 3 degrees of freedom, where N is the total number of the cross-validation groups from all batches included in the study.

## Results and discussion

In the following sections we apply the strategy presented in Fig. 1 to study the SLE samples from the Karolinska SLE cohort.

### Representative selection: sample selection and subdivision into batches based on clinical and personal data

**Fig. 2:**
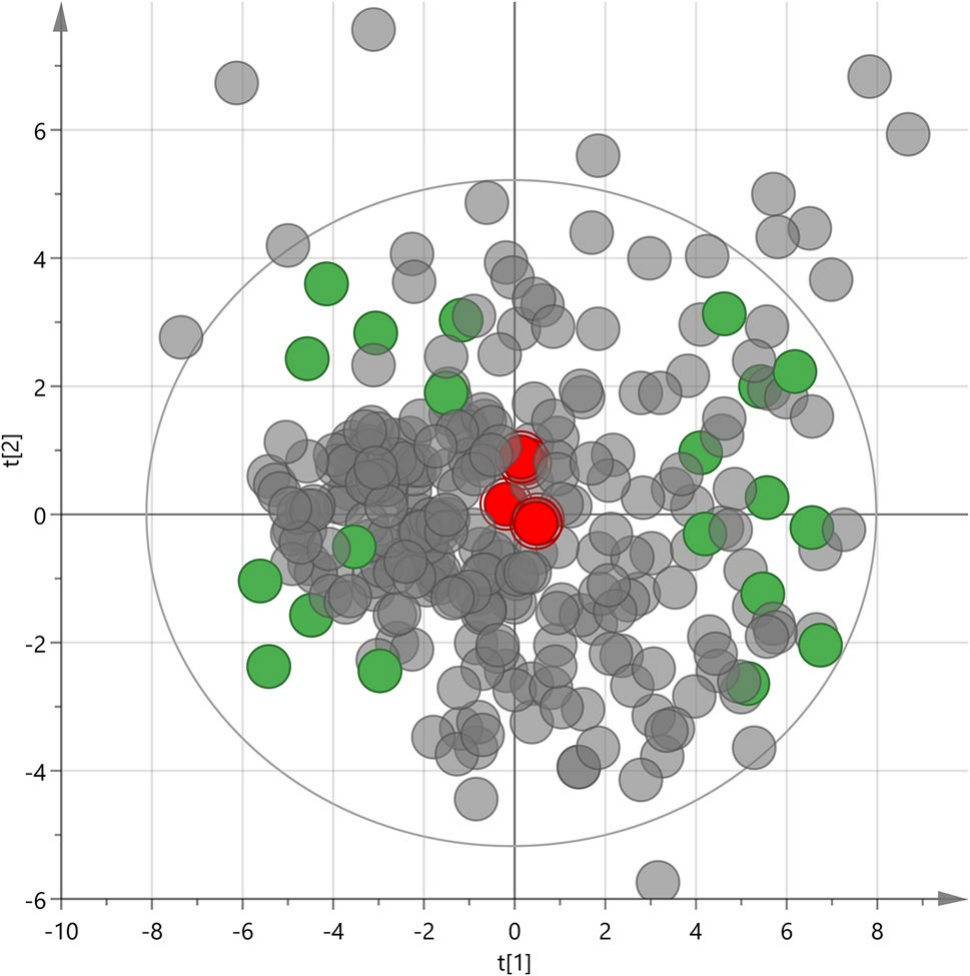
Sample selection from controls. From the PCA model (R2X[1] = 0.107, R2X[2] = 0.044), 20 samples from the full two-level factorial design corners (5 from each) + 3 center points were selected so that they spanned the entire multivariate space defined by samples and their associated clinical data. Selected samples are marked in *green* (design corners) and *red* (center points); samples that were not selected are marked in *gray*

Patients’ personal and clinical data were used to calculate separate PCA models for each of the five subgroups and the control group. In our study, from each PCA model, 23 samples were selected so that they spanned the multivariate space defined by all the samples and their associated (available) clinical descriptors (see Sect. 2). Figure 2 shows sample selection from the control group as an example of the applied sampling procedure. Fig. S1 shows the selection principle. Two principal components were used since they accounted for the highest amount of variation in the data, with third component in most cases being not significant according to the cross-validation procedure. Other components, if significant, could be also used and subset selection could be performed for example with the application of the generalized subset designs (Surowiec et al. 2017). Obtaining a perfect design fit (for example square for the two level two factors full factorial design) for the PCA score plot is not always possible, especially if many samples (relative to all samples available) are taken at each of the design points. However, the goal of representative selection is to be as close to the selected design as possible.

The size of the virtual square (full two-level factorial design) that should fit into the score plot is based on whether a smaller space with less variation (smaller square) or one covering wider variation within the samples available (wider square) is desired. The smaller square removes all outliers that could introduce unwanted variation in the subsequent data analysis, and is therefore the more optimal approach for pilot and exploratory studies. The wider square represents a wider spread of variation in the data and is better suited for more comprehensive studies with larger amount of analyzed samples (Fig. S1).

The samples were further separated into four batches using the same approach described above because of the limited, maximum, daily sample throughput of the GC–TOF–MS instrument. Five to six samples from each subgroup and the control group were analyzed in each batch. An uncontrollable drop in instrument’s sensitivity during the analysis was observed for batch 4, which was therefore excluded from further analysis.

The selection method we used ensured that samples represented the multivariate space defined as samples and their related descriptors and hence were representative for the studied cohort. Dividing the samples into four batches representatively allowed for treatment of each batch as an individual study and gave the basis for independent data analysis. This approach provided full control over the analytical pipeline and ensured that, despite the exclusion of one batch from study analysis due to problems with instrument stability, remaining batches still carried information required by the study. If the samples had not been divided in a controlled manner, loss of samples due to uncontrollable factors could result in the need for a whole new analysis.

### Analytical data evaluation of the SLE multi-batch data

To get a general overview and understanding of the spread of variability in the data, we performed combined analysis of all batches with PCA. In our study, the score plot from the PCA model of normalized data from three batches (4 components, 105 samples, 73 variables, R^2^X = 0.4) revealed sample separation based on day of analysis as the main source of variation in the data (Fig. S2). This finding suggested the need for individual analysis of each batch. Here we present multivariate approach for performing results compilation from different batches. We have focused on the differences between SLE patients and controls, since this was the first aim of the study, with the main assumption being that analysis of the metabolic profile differentiating SLE patients from the control would improve both disease diagnosis, as well as understanding of its pathology. With the balanced and representative number of samples from each disease sub-class, finding metabolic profile characterizing SLE is expected to be a reliable approach.

We applied OPLS-DA modelling to evaluate differences between SLE and control. OPLS-DA models were created for each individual batch. Table 1 provides the parameters of the models studied and the p(corr) values obtained are presented in Table 2. Significance of the metabolites according to jackknife confidence intervals in individual OPLS-DA models varied between batches, with twenty-one compounds being significant in at least one batch, two of them (ornithine and tryptophan) significant in two batches and one (2-oxoisocaproic acid) significant in all batches.

**Table 1:** Parameters of the OPLS-DA models used to discriminate between the SLE and population-based control groups

Batch	A	p(1) (%)	N	R^2^X	R^2^ (cum)	Q^2^	CV-ANOVA
Batch 1	1 + 1 + 0	9.1	35	0.24	0.75	0.42	p = 0.002
Batch 2	1 + 0 + 0	7.3	36	0.07	0.52	−0.10	p = 1
Batch 3	1 + 1 + 0	6.0	34	0.18	0.81	0.42	p = 0.003

**Table 2:** P(corr) loadings from the OPLS-DA models between SLE and control groups in each batch and in averaged profile

Compound name	HMDB	Compound class	Batch 1	Batch 2	Batch 3	Average p(corr) value	Average confidence interval
1-5-Anhydro-d-glucitol	HMDB03911	Carbohydrate	0.324	−0.106	−0.045		
2-Oxoisocaproic acid	HMDB00695	Organic acid	−0.560	−0.527	−0.652	−0.579	0.387
3-Hydroxybutanoic acid	HMDB00357	Organic acid	−0.308	−0.122	−0.104	−0.178	0.614
4-Hydroxyphenylacetic acid	HMDB00020	Organic acid	0.212	0.196	0.078	0.162	0.340
Adenosine-5-monophosphate	HMDB00045	Nucleotide	−0.318	−0.395	−0.039	−0.251	0.497
Alanine	HMDB00161	Amino acid	0.150	0.267	−0.335		
Allothreonine	HMDB60878	Amino acid	0.126	0.251	−0.492		
α-Aminobutyric acid	HMDB00452	Organic acid	−0.027	−0.080	−0.350	−0.152	0.412
α-Ketoglutaric acid	HMDB00208	Organic acid	0.297	−0.104	0.169		
α-Linolenic acid (ALA)	HMDB02181	Fatty acid	−0.647	−0.268	−0.159	−0.358	0.684
α-Tocopherol	HMDB01893	Sterol	0.048	−0.088	0.046		
Arachidonic acid	HMDB01043	Fatty acid	−0.411	−0.343	−0.176	−0.310	0.526
Arginine	HMDB00517	Amino acid	0.382	0.320	0.147	0.283	0.352
Asparagine	HMDB00168	Amino acid	0.090	0.098	−0.293		
β-Sitosterol	HMDB00852	Sterol	0.100	0.050	0.207	0.119	0.383
Caffeine	HMDB01847	Nucleotide	0.105	−0.182	0.138		
Campesterol	HMDB02869	Sterol	0.247	0.050	0.091	0.129	0.418
Cholesterol	HMDB00067	Sterol	0.004	0.090	0.004	0.033	0.465
Citric acid	HMDB00094	Organic acid	−0.189	−0.183	−0.315	−0.229	0.498
Creatinine	HMDB00562	Amino ketone	0.384	0.241	0.201	0.275	0.388
Cystathionine	HMDB00099	Amino acid	0.216	0.161	0.087	0.155	0.370
Cysteine	HMDB00574	Amino acid	−0.275	−0.050	−0.124	−0.150	0.548
Cystine	HMDB00192	Amino acid	0.313	0.409	0.156	0.293	0.379
Docosahexaenoic acid (DHA)	HMDB03581	Fatty acid	−0.292	−0.540	−0.201	−0.344	0.565
Elaidic acid	HMDB00573	Fatty acid	−0.682	−0.301	−0.178	−0.387	0.626
Erythronic acid	HMDB00613	Carbohydrate	−0.043	−0.267	0.258		
Galactitol	HMDB00107	Carbohydrate	0.388	0.317	0.103	0.269	0.430
γ-Tocopherol	HMDB01492	Sterol	−0.152	0.332	0.180		
Gluconic acid	HMDB00625	Organic acid	−0.029	−0.034	−0.514	−0.193	0.363
Glucosamine	HMDB01514	Carbohydrate	−0.010	0.051	−0.280		
Glucose	HMDB00122	Carbohydrate	−0.403	−0.108	−0.307	−0.272	0.335
Glutamic acid	HMDB00148	Amino acid	0.096	0.056	0.280	0.144	0.259
Glutamine	HMDB00641	Amino acid	0.176	0.338	0.108	0.208	0.282
Glyceric acid	HMDB00139	Organic acid	0.388	0.023	0.210	0.207	0.445
Glycerol	HMDB00131	Polyol	−0.122	−0.218	0.246		
Glycerol-3-phosphate	HMDB35909	Organic acid	0.154	−0.041	0.248		
Glycine	HMDB00123	Amino acid	0.264	0.256	0.097	0.206	0.340
Hexadecanoic acid	HMDB00220	Fatty acid	−0.640	−0.385	−0.278	−0.434	0.620
Hippuric acid	HMDB00714	Amino acid	0.299	−0.194	0.332		
Histidine	HMDB00177	Amino acid	−0.045	0.185	−0.633		
Inosine	HMDB00195	Nucleoside	−0.104	−0.370	−0.203	−0.225	0.535
Lactic acid	HMDB00190	Organic acid	0.159	0.037	0.034	0.077	0.361
Lactose	HMDB00186	Carbohydrate	−0.418	0.240	0.235		
Lauric acid	HMDB00638	Fatty acid	−0.550	−0.236	−0.133	−0.307	0.391
Linoleic acid	HMDB00673	Fatty acid	−0.509	−0.233	−0.330	−0.357	0.698
Lysine	HMDB00182	Amino acid	0.162	0.347	−0.371		
Malic acid	HMDB00156	Organic acid	0.114	−0.216	−0.037		
Maltose	HMDB00163	Carbohydrate	−0.632	−0.307	−0.013	−0.317	0.637
Methionine	HMDB00696	Amino acid	0.170	0.174	−0.445		
Methyl linoleate	HMDB34381	Fatty acid methyl ester	0.391	0.162	0.289	0.281	0.313
Nonanoic acid	HMDB00847	Fatty acid	−0.039	−0.066	0.057		
*o*-Phosphoethanolamine	HMDB00224	Organic phosphoric acid	−0.391	−0.341	−0.060	−0.264	0.475
Ornithine	HMDB00214	Amino acid	0.431	0.489	0.093	0.338	0.323
Oxalic acid	HMDB02329	Organic acid	−0.218	0.180	0.033		
Palmitoleic acid	HMDB03229	Fatty acid	0.289	0.205	−0.037		
Phenylalanine	HMDB00159	Amino acid	0.062	−0.021	−0.212		
Phosphoric acid	HMDB02142	Inorganic acid	−0.105	−0.032	0.209		
Proline	HMDB00162	Amino acid	0.102	0.476	−0.065		
Pyroglutamic acid	HMDB00267	Amino acid	−0.053	0.215	0.181		
Scyllo-inositol	HMDB06088	Polyol	0.275	0.312	0.085	0.224	0.410
Serine	HMDB00187	Amino acid	0.192	0.069	0.254	0.172	0.240
Squalene	HMDB00256	Carbohydrate	−0.114	0.336	0.015		
Stearic acid	HMDB00827	Fatty acid	−0.592	−0.495	−0.073	−0.387	0.497
Sucrose	HMDB00258	Carbohydrate	0.165	0.355	0.040	0.187	0.407
Taurine	HMDB00251	Amino acid	−0.340	−0.577	0.103		
Threonic acid	HMDB00943	Organic acid	−0.003	−0.050	0.262		
Threonine	HMDB00167	Amino acid	0.251	−0.130	0.332		
Tryptophan	HMDB00929	Amino acid	−0.197	−0.364	−0.448	−0.336	0.258
Tyrosine	HMDB00158	Amino acid	0.097	0.242	−0.118		
Uric acid	HMDB00289	Purine	0.309	0.250	0.186	0.248	0.508
Valine	HMDB00883	Amino acid	−0.168	0.022	−0.436		
Xylitol	HMDB02917	Polyol	0.430	0.416	0.054	0.300	0.385
Xylose	HMDB00098	Carbohydrate	0.180	0.475	0.238	0.298	0.304

Underlined metabolites significant according to jackknifing. Positive values represent metabolite increased in SLE patients compared to the population-based controls

**Fig. 3:**
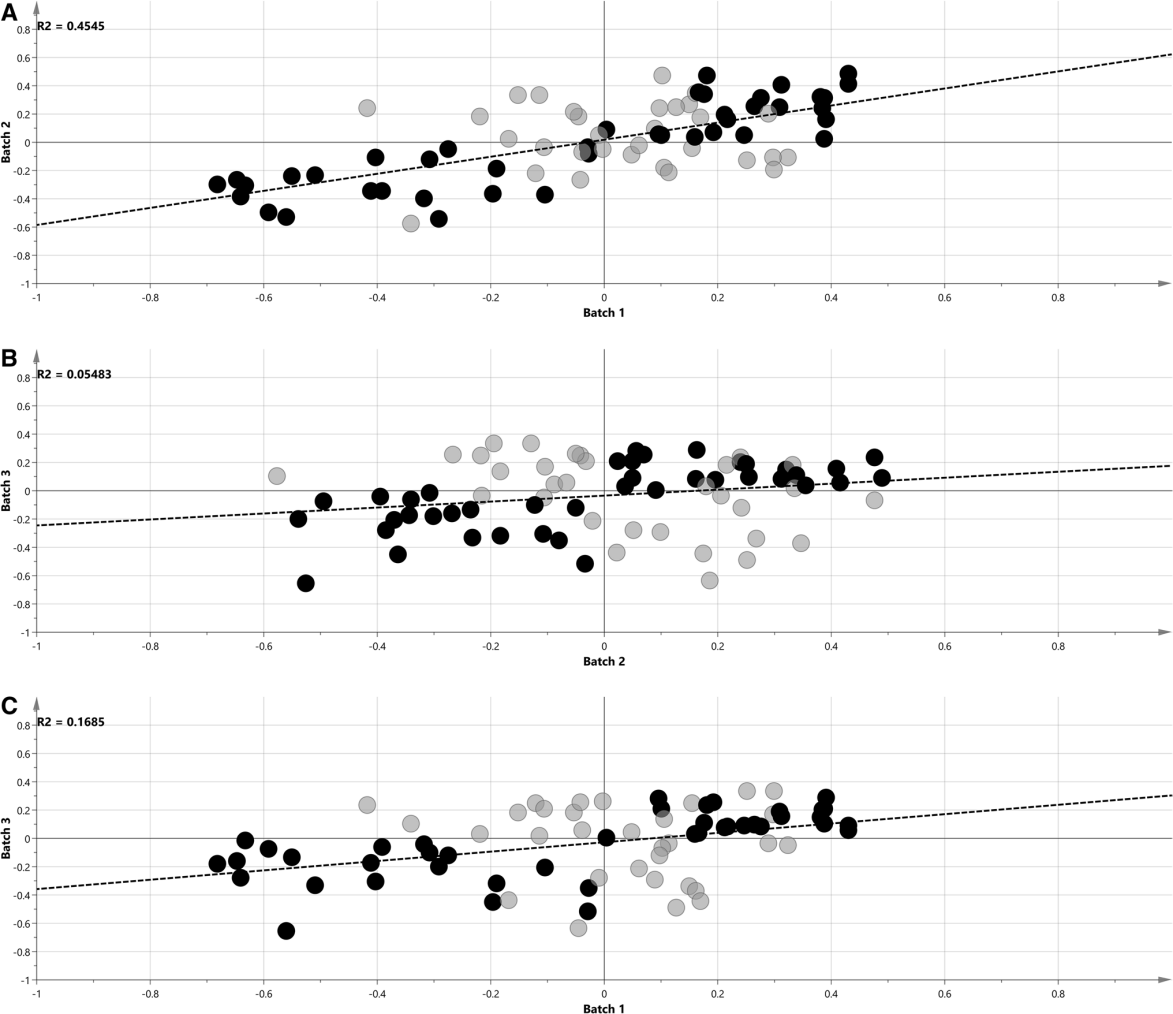
SUS plot analysis of p(corr) vectors from the first and second batch (**a**), the second and third batch (**b**), and the third and first batch (**c**). Metabolites with the same change direction from all batches studied are indicated in *black*; the *dashed line* is the regression line; *R* ^2^ regression coefficient

To compare similarity of metabolic profiles between batches, we further investigated shared and unique structure plots (SUS-plots) (Wiklund et al. 2008) with p(corr) vectors from the OPLS-DA models between SLE and control groups for each batch (Fig. 3). For identical profiles, the SUS-plot should have all the points on the diagonal line from the lower left corner to the upper right corner, with R^2^ = 1.0. Figure 3 shows that the correlation between p(corr) vectors from each batch was low, and this was confirmed when correlation coefficients were calculated (R^2^ = 0.454 for Batch 1 and Batch 2, R^2^ = 0.177 for Batch 1 and Batch 3, and R^2^ = 0.055 for Batch 2 and Batch 3). A low correlation between p(corr) vectors showed that there were no strong metabolic differences between SLE and controls, what was especially seen in the weak model for the Batch 2. Still, obtaining a common metabolic profile could give relevant information about perturbations in metabolite levels between SLE and controls.

**Fig. 4:**
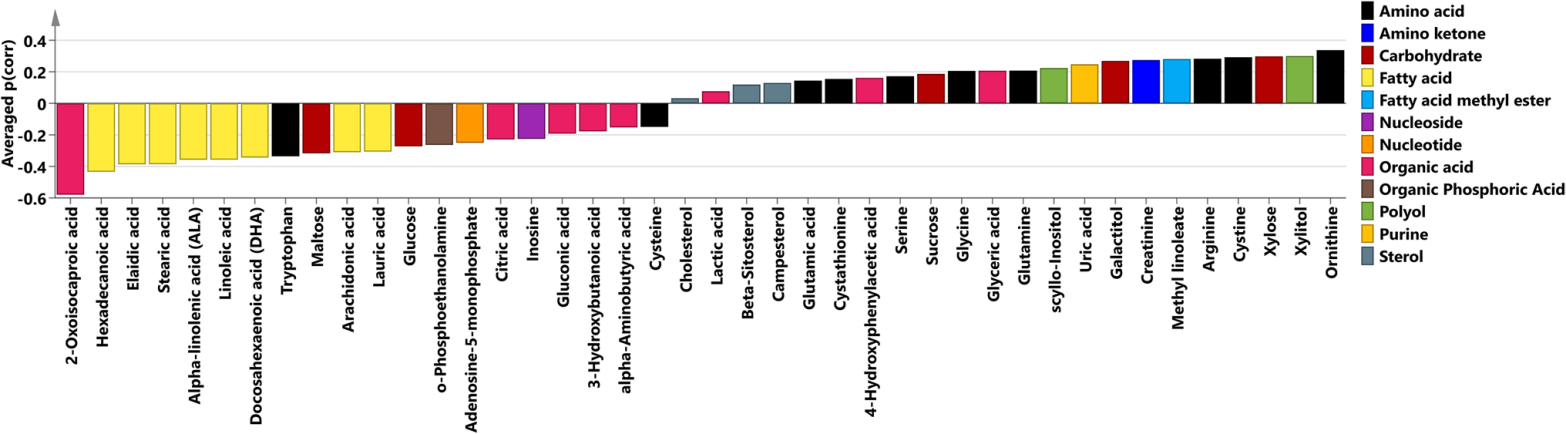
Combined metabolic profile of SLE versus controls. The p(corr) value presented is the average p(corr) value of the three batches for the metabolites that showed the same change direction relative to SLE in all batches studied

After further SUS-plot analysis, forty two compounds with the same change direction [sign of p(corr) vector] in all the OPLS-DA models were selected. These metabolites were considered reliable, and formed a combined metabolic profile describing differences between SLE patients and controls. Their p(corr) values and jackknife standard errors were used to calculate the average p(corr) values and corresponding confidence intervals respectively, as summarized in Table 2. Three metabolites had averaged confidence intervals that were lower than the absolute value of the average p(corr) value (2-oxoisocaproic acid, ornithine and tryptophan), and these metabolites were considered significant in the combined profile differentiating SLE patients from controls which is portrayed in Fig. 4. This profile was further used for elucidation of metabolic pathways that could be disturbed in SLE.

### Biological relevance of the SLE versus controls metabolic profile

In multivariate approach interpretation of the metabolic profile is based on an assumption that if metabolites involved in a certain metabolic pathway demonstrate changes that would not be anticipated by random chance, then this pathway is probably biologically or metabolically important. This assumption is valid even if a few single metabolites do not show significant changes.

Our study showed decrease in levels of most free, long-chain fatty acids in SLE patients compared to controls. These results confirm the ones presented by Wu et al. (Wu et al. 2012), who connected this change to increased β-oxidation in SLE patients. Reduced fatty acid levels could also indicate decreased lipolysis in SLE patients (Borba et al. 2000). Reduction of levels of unsaturated fatty acids could, on the other hand, result from accelerated lipid peroxidation, as previously demonstrated in SLE (Frostegard et al. 2005), or from increased synthesis of inflammatory mediators which are products of polyunsaturated fatty acid oxidation (Dennis and Norris 2015).

In the averaged metabolic profile we obtained, SLE patients had higher levels of most amino acids in their plasma, except for: cysteine and tryptophan, which was significant according to jackknifing. This contradicts findings described by others (Ouyang et al. 2011). Lower levels of tryptophan were observed in SLE patients before (Bengtsson et al. 2016) and could be connected to changes in kynurenine pathway and activation of immune response (Lood et al. 2015; Perl et al. 2015). Arginine and ornithine had higher, although not significant according to jackknifing, levels in SLE patients compared to controls, which have been also reported by others (Wu et al. 2012). This finding could be related to nitrogen oxide (NO) production and to urea cycle disorders, since the urea cycle is the sole source of endogenous arginine, ornithine, and citrulline in humans. Increased NO synthase activity has already been associated with SLE (Wigand et al. 1997). In general, understanding the role of amino acids in SLE requires more effort.

## Conclusions

In this study we presented a strategy for representative sample selection and OPLS-based integration of results from multi-batch experiments in metabolomics. We applied this strategy in the clinical study of SLE. Design of experiment-based sample selection allowed obtaining a representative subset of samples spanning all the physicochemical variability contained within the cohort studied, defined by the available samples along with their associated personal and clinical descriptors. Presented approach is valid for controlled selection of subgroups of samples from larger cohorts in which samples are characterized by number of clinical, personal, environmental etc. parameters. It is also applicable for representative division of such samples into smaller groups for chemical analysis in situations where all samples cannot be analyzed concurrently. Controlled sample selection reduces the risk of bias and is a first step towards obtaining reliable and robust results.

Profiling data from each batch of samples were analyzed separately with application of OPLS modelling. Obtained metabolic profiles in form of p(corr) vectors were subsequently averaged to provide combined metabolic profile differentiating SLE patients from controls, which was later evaluated in relation to metabolic pathways that could be disturbed in SLE.

Because of the applied methodology, which was based on strict control of each experimental step, we were able to obtain a reliable metabolic profile that characterized the comparison between SLE patients and controls. The work presented in this paper emphasizes the importance of applying multivariate approaches for representative sample selection and subsequent integration of ‘omics’ results obtained from different analytical batches. The applied data analysis approach may be used for compilation of results from different analytical batches and for combination of results from different studies. We believe that this methodology will lead to more reliable results and will enable not only comparison of data analyzed at different times, but also ones obtained from different research groups.

## Electronic supplementary material

Below is the link to the electronic supplementary material.

Supplementary material 1 (DOCX 217 KB)
