# Supplementary material for: Multivariate strategy for the sample selection and integration of multi-batch data in metabolomics
Source: Metabolomics. 2017 Aug 24;13(10):114. doi: 10.1007/s11306-017-1248-1 (PMC5570768; doi:10.1007/s11306-017-1248-1)
Supplement: Supplementary file 1 — Supplementary material 1 (DOCX 217 KB) [file 11306_2017_1248_MOESM1_ESM.docx]

**Multivariate strategy for the sample selection and integration of multi-batch data in metabolomics**

**Supplementary Material**

Izabella Surowiec^1^, Erik Johansson^2^, Frida Torell^1^, Helena Idborg^3^, Elisabet Svenungsson^3^, Per-Johan Jakobsson^3^, Johan Trygg^1,2^*

1. Computational Life Science Cluster (CLiC), Department of Chemistry, Umeå University, 901 81 Umeå, Sweden
2. MKS Data Analytics Solutions, 907 19 Umeå, Sweden
3. Rheumatology Unit, Department of Medicine, Solna, Karolinska Institutet, Karolinska University Hospital, 171 76 Stockholm, Sweden

(*) – **Corresponding** author: Johan Trygg, PhD, johan.trygg@umu.se; **Telephone:** +46 **730647137; Address**: Department of Chemistry, Umeå University, Linnaeus väg 10, 901 87 Umeå, Sweden

**Supplementary Methods**

*Metabolite extraction from plasma*

Frozen plasma was thawed at room temperature for 10 min and stored on ice (4^o^C). Plasma samples were prepared for GC-TOF-MS analysis by adding 720 µl extraction mix (methanol:H2O, 9:1, v/v), containing all eleven isotopically labelled internal standards (7 ng/µl), to 80 µl aliquots of plasma. Each sample was extracted vigorously using a MM 400 Vibration Mill (Retsch GmbH & Co. KG, Haan, Germany) at a frequency of 30 Hz for 3 min, followed by centrifugation at 18 620 g for 15 min at 4^o^C. A volume of 200 µl supernatant was transferred to a GC vial and evaporated to dryness in a miVac QUATTRO concentrator (Genevac LTD, Ipswich, UK) for approximately 2h at room temperature.

*Derivatization of samples*

A 30 µl of methoxyamine (15 µg/µl) in pyridine was added to each GC vial and the resultant mixture was shaken vigorously for 10 min. Methoxymation was performed at room temperature for 16 h, followed by the addition of 30 µl MSTFA with 1% TMCS to each sample (brief vortex). Samples were left at room temperature for 1 h to allow silylation to occur, followed by the addition of 30 µl heptane (containing 15 ng/µl methyl stearate as an internal standard) and a brief vortex for 10 s.

*GC-TOF-MS analysis*

A volume of 1 µl of each derivatzed sample was injected splitless by a CTC Combi Pal autosampler (CTC Analytics AG, Zwingen, Switzerland) into an Agilent 7890 GC equipped with a 30 m x 0.18 mm i.d. fused-silica capillary column chemically bonded with 0.25-um DB 5-MS stationary phase (J&W Scientific Folsom, CA). The injector temperature was set to 260^o^C. Helium was used as the carrier gas at a constant flow rate of 1 mL min-1 through the column. For every analysis, the purge time was set to 75 s at a purge flow rate of 20 mL/min and an equilibrium time of 1 min. The column temperature was held initially at 70^o^C for 2 min, then increased to 320^o^C at a rate of 20^o^C/min, where it was held for 12 min. The column effluent was introduced into the ion source of a Pegasus III time-of-flight mass spectrometer (Leco Corp., St Joseph, MI, USA). The ion source and transfer line temperatures were set to 200^o^C and 250^o^C, respectively. Ions were generated by a 70 eV electron beam at a current of 2.0 mA. Masses were acquired in the mass range 50-800 m/z at a rate of 20 spectra/s. The acceleration voltage was turned on after a solvent delay of 150 s. The detector voltage was 1670 V.

An alkane series (C10-C40) was run together with all samples to enable calculation of retention indices (RI) of the compounds. Quality controls samples created from pooled plasma from the studied samples were extracted and run together with the rest of the samples in the randomized order (every 5 to 10 injections) to control stability of the analysis over time.


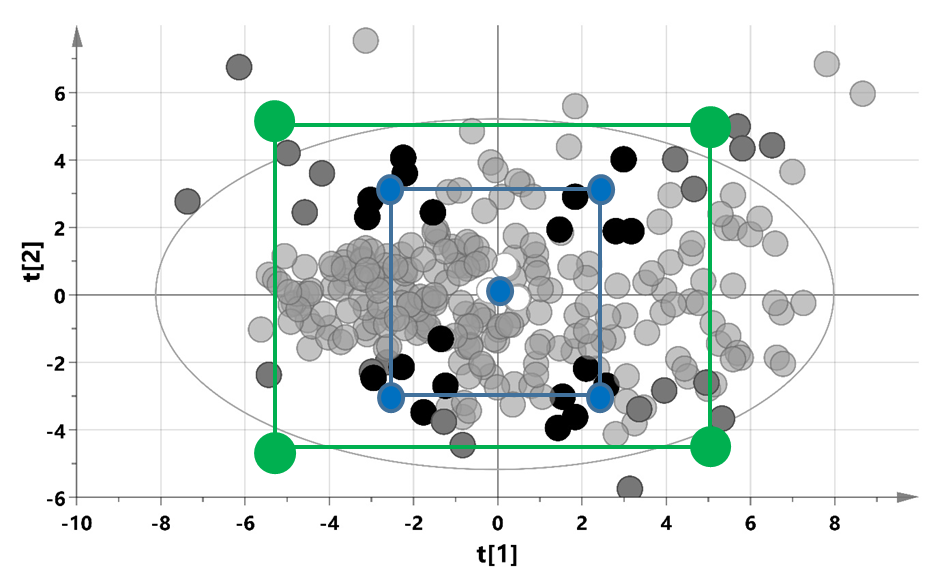


**Fig. S1.** Schematic representation of sample selection for the two level two factors full factorial design. Samples were selected from all available samples (light gray circles) and their associated clinical descriptors, using a PCA model, according to the full factorial design with two factors and a center point (blue square and circles). Five samples were selected from the design corners (black circles), and three samples from nearby the design center point (white circles). Alternative selection covering wider spread of variation in the data is presented as the green square with the selected samples in dark gray color.

This sample selection method was repeated for each SLE subgroup and the control group and ensured that selected samples spanned the whole multivariate space defined by the samples and their associated clinical data.

**Fig. S2.** PCA plot of all acquired metabolomics data, with samples from each batch marked in a different color.
